# How to cope with Long COVID – A qualitative interview study on stressors and coping strategies of people affected by long-term consequences of COVID-19

**DOI:** 10.1371/journal.pone.0343115

**Published:** 2026-02-23

**Authors:** Melanie Elgner, Marius Binneböse, Josi Großmann, Tamara Frank, Paul Bruckmann, Claas Lahmann, Katrin Elisabeth Giel, Christine Allwang, Florian Junne, Hannah Wallis

**Affiliations:** 1 University Clinic for Psychosomatic Medicine and Psychotherapy, University Medicine, Otto-von-Guericke-University Magdeburg, Medical Faculty, Magdeburg, Germany; 2 German Center for Mental Health (DZPG), Partner Site Halle-Jena-Magdeburg, Magdeburg, Germany; 3 Department of Psychosomatic Medicine and Psychotherapy, TUM University Hospital, School of Medicine and Health, Technical University of Munich, Munich, Germany; 4 Department of Psychosomatic Medicine and Psychotherapy, University Hospital Freiburg, Freiburg, Germany; 5 Department of Psychosomatic Medicine and Psychotherapy, Medical University Hospital Tübingen, Tübingen, Germany; 6 German Center for Mental Health (DZPG), Partner Site Tübingen, Tübingen, Germany; 7 Center for Behavioral Brain Sciences (CBBS), Magdeburg, Germany; PLOS: Public Library of Science, UNITED KINGDOM OF GREAT BRITAIN AND NORTHERN IRELAND

## Abstract

Long COVID, a multi-system-disease characterized by persistent somatic and mental symptoms following a SARS-CoV-2 infection, can severely impair health and quality of life of those affected. In the absence of adequate therapeutic approaches and a fragmented care landscape, our focus is on identifying individual stressors, the resulting needs and strategies people use to cope with the ongoing burden of the disease and its long-term stressors. This qualitative interview study is part of a pilot multicenter study addressing psychosocial needs in patients with Long COVID. The surveyed sample (n = 40) consists of affected people, who suffer from persistent symptoms and psychosocial stress after a SARS-CoV-2 infection. Based on the Transactional Stress Model according to Lazarus and Folkman and the Brief COPE by Carver, the qualitative analysis of semi-structured interviews focused on the various and individual coping attempts of the interviewees. Participants reported a wide range of persistent physical and mental complaints. Fatigue-associated complaints, cognitive impairments, fears and worries were mentioned frequently and perceived as particularly stressful. Job insecurity and financial worries, lack of recognition, stigmatization, lack of treatment and therapy approaches, withdrawal and social isolation were reported as stressors. In most cases, we identified an interplay between emotion-oriented (such as emotional support, self-care and positive thinking) and problem-oriented coping strategies (such as planning/pacing, self-help, withdrawal and avoidance). Emotional support as the most frequently mentioned strategy and as a fundamental resource in coping with this disease should be strengthened. These findings offer a valuable insight into the diverse stressors and coping patterns in dealing with post-viral symptoms of COVID-19. The analysis reveals that complaints and attempts to cope vary significantly among the participants. This underlines the importance of providing tailored support to those affected to help them manage their symptoms, improve their quality of life and enable them to participate in social life again.

## Introduction

After the worldwide pandemic of COVID-19, the biggest public health crisis in the 21st century, it is assumed that around 10–20 percent of people develop persistent symptoms after an acute SARS-CoV-2 infection [1]. The World Health Organization (WHO) defines Long COVID as post COVID-19 condition, whereby symptoms usually start within three month after the SARS-CoV-2 infection, persist for at least two months and cannot be explained by any other condition. Symptoms can persist after the infection, fluctuate in their intensity or even reoccur after a symptom-free period. According to the WHO, symptoms can last from weeks to months to years [1]. It is not yet possible to predict exactly how long the condition will last. With over 200 different symptoms reported, common symptoms include fatigue or rapid exhaustion, difficulties with breathing, cognitive impairments and various types of pain. Fatigue has been identified as the main symptom [2]. In addition to the potentially numerous and severe physical symptoms, psychological symptoms should not be underestimated either. A systematic review and other studies showed a significant burden due to mental health issues, including depression, anxiety, stress and sleep disturbances in people recovering from COVID-19 [3–5]. Persistent somatic and psychological symptoms following a SARS-CoV-2 infection can affect all areas of life – everyday activities such as work, leisure time and social life – and can result in serious health restrictions with a drastic effect on the quality of life and social participation of those affected [6–9]. Furthermore, there are various barriers in treatment and the healthcare system [10,11]. There is a lack of specialized care, an urgent need to improve treatment, social and financial support and social representation of Long COVID [12]. Patients suffering from Long COVID, therefore, have a variety of different symptoms on a physical, psychological and social level [13].

The underlying causes of these long-term consequences of COVID-19 still remain limited. There are no adequate therapeutic approaches or proven drug treatments yet. Long COVID therefore remains a clinical challenge for physicians and mental health practitioners alike [14]. For people affected by long-term consequences of COVID-19, dealing with persistent symptoms requires a great amount of adjustment. Currently, the most common approach is to help those affected to improve their quality of life by regaining physical, cognitive and emotional functions [1]. As long as there is no causal treatment for the physical symptoms and due to the growing impact on mental health, we lay focus on psychological and psychosocial approaches with the need for a tailored intervention and comprehensive psychosocial support [3,15]. Within the *PsyLoCo* pilot-study (“Psychosocial needs in patients with Long COVID”) a psychosomatic-psychotherapeutic intervention was developed by an interdisciplinary project team to specifically address the psychosocial needs of people affected by Long COVID [16,17]. One part of the development of this intervention is the qualitative interview study with Long COVID patients regarding their psychosocial needs, preferences and coping strategies.

Previous studies have focused specifically on coping with the COVID-19 pandemic. A systematic review shows that active coping strategies (e.g., planning) and avoidance strategies increased during the pandemic [18]. Furthermore, there has been an increase in effective coping strategies, confirming that people seek alternative ways to satisfy their basic needs when these are compromised over a longer period of time, for example due to a lockdown. Social support, family support and participation in sporting activities were identified among students as coping strategies that helped to mitigate the impact of the pandemic on their mental health. Alcohol consumption and smoking worsened their mental health [19]. Among nurses, one coping strategy to prevent the development of mental disorders during the pandemic was mindfulness [20] and among healthcare professionals problem-oriented strategies were most commonly used in coping with stress [21]. In general, a need for strategies to support people with lower coping skills is suggested [22].

In contrast to coping strategies regarding the pandemic, there are fewer publications on coping strategies in connection with Long COVID. When dealing with the loss of smell and taste after COVID-19, practical and emotional coping strategies and the profound emotional and psychological impact are outlined [23]. When coping with pain after COVID-19, pharmacological measures, emotional/cognitive aspects (i.e., psychological and/or coping strategies) and exercise programs are involved [24]. Female long haulers tend to cope with cognitive and emotional strategies (e.g., increasing knowledge, planning, realistic goal setting, emotions management), behavioral strategies (e.g., physical activity, healthier eating habits), spiritual strategies (e.g., connecting with God) and social support (e.g., support from family and friends, co-workers, therapists and counselors, online support groups with other patients) [25]. These findings confirm the prominent role of family, friends and support groups as powerful resilience resources. In a qualitative study of 47 adults, some respondents mentioned support from friends, family and the community as helpful for their coping experiences (social support) [6]. Former COVID-19 inpatients also focused on social, family and community support during their recovery [26]. Patients with persistent symptoms often resort to withdrawal and emotion-focused coping strategies, indicating an increased need for psychosocial support [8]. In general, it has been shown that further research is needed to examine the psychosocial impacts of Long COVID and psychosocial resources are important for coping with Long COVID [27]. In particular, social support – including family and friends – has been shown to be an important protective factor [28,29]. Another study shows an improvement in condition of Long COVID sufferers through group meetings with the support of psychotherapists and occupational therapy [11]. However, little research has been conducted into whether and how these strategies affect complaints in the long term [30].

Besides physical and mental complaints, there are further pervasive effects after a SARS-CoV-2 infection. Specific stressors for people affected by Long COVID have so far been described as socioeconomic struggles or financial difficulties and psychosocial restrictions. These include loss of employment or an impaired ability to perform required tasks, social and educational setbacks, insurance loopholes, isolation, skepticism and resistance from others, social stigmatization, identity shifts or loss, grief of losing meaningful roles, loss of independence and envisioned futures [12,26,27,31]. Financial challenges were identified as a key issue. Physical and cognitive stressors can lead to a worsening of symptoms, underscoring the need for effective therapeutic strategies and interventional trials [31].

This qualitative study addresses the question of how people affected by the long-term consequences of COVID-19 manage their symptoms and their individual psychosocial stress and what care needs result from this in order to reduce the distress and burden of the illness. Based on the results of the conducted interviews, this article focuses on identifying individual stress factors, the resulting needs and requirements, and the strategies used by those affected to cope with the ongoing burden of the illness and its long-term stressors. In the absence of a treatment or cure for Long COVID, the focus is on how those affected are coping with the disease and which methods have shown a positive effect so far. Therefore, coping with symptoms is one important component and a specific module of the *PsyLoCo* intervention [17]. This may also be a crucial factor in future pandemics and other post-viral phenomena with long-term consequences.

## Methods

### Study design

This qualitative interview study was part of the *PsyLoCo* study, a multi-center pilot-study to develop a specialized modular intervention addressing psychosocial needs in patients with Long COVID [16,17]. The pilot-study was conducted as a randomized and controlled study at six locations across Germany. Participating locations in the interview study were Magdeburg and Munich. In dialog with those affected, individual experiences and complaints associated with Long COVID were recorded. In addition to the Long COVID symptoms, participants were also asked about their personal burden and stress levels caused by the disease. A particular focus here was on the psychological and social (psychosocial) needs of the patients, the need for supply and the options for help in dealing with the disease. The interview data provided a basis for a psychotherapeutic manual that is specifically designed for the treatment of patients with Long COVID [17].

### Data collection

We recruited persons to participate in individual interviews from July to September 2022 via the psychosomatic outpatient clinics in Magdeburg and Munich. We also used flyers, press releases, social media, as well as telephone and email contact to encourage people to participate in the study. Eligibility criteria for participants were as follows. Participants should be 18 years or older to be included. A previous SARS-CoV-2 infection should be confirmed by either a PCR-test result or a rapid self-test (self-disclosure by participants). Participants should be able to report at least one persistent symptom (lasting at least four weeks after the initial infection, regarding to the definition of Long COVID in the German S1 guideline long/post-COVID syndrome [32]) and they should be able to give informed consent. The exclusion criterion was the presence of a severe mental health disorder according to the ICD-10 sections F10-19 (Mental and behavioural disorders due to psychoactive substance use) and F20-29 (Schizophrenia, schizotypal and delusional disorders) [16]. Before the interview started, each participant had to sign a declaration of consent and received detailed information about the study, data security and their rights as participants. Possible side effects as well as benefits were explained. All interviewees gave both oral and written consent and a physical or electronic signature was obtained.

### Data processing

We used a semi-structured interview guide, outlined in Table 1, to collect individual experiences, complaints, personal burdens, psychosocial needs and coping strategies associated with Long COVID. The interview duration was set to 30–45 minutes. The guideline was drawn up by the interdisciplinary study group based on Helfferich [33] and the German S1 guideline long/post-COVID syndrome [32], which was adapted through a regular discussion and synthesis process. A trial interview was held at each location. Interviews were conducted, mostly via telephone and recorded using iPads or other dictation devices. We conducted all interviews within an 8-week period. The audio recordings were transcribed and anonymized by a professional transcription agency.

**Table 1:** Interview Guide (extract).

Topic	Question	Focus
General physical health	“How have you been physically since the infection, have you experienced any persistent symptoms/complaints?” “How do you cope or deal with your physical complaints?”	Physical symptoms after COVID-19
Pre-existing physical or mental illnesses/conditions	“Have you had any severe physical or mental symptoms/complaints before being infected by COVID-19?”	Symptoms before COVID-19-infection and COVID related changes
General mental health	“How have you been mentally since the infection?”	Long COVID mental ailments
Lay genesis	“What do you believe caused your symptoms/ailments?”	Individual theories and subject models regarding illness
Everyday life and social life	“How do the symptoms affect your everyday life?”	Daily impairments and coping
Work life	“Have you experienced any problems at work?” “Have you returned to your job?”	(In)ability to work or return to work
Coping	“Overall, what has helped you the most in coping with the long-term effects of COVID-19?”	Greatest help in dealing with complaints, stress, and emotions
Treatment and Support	“Which treatments did you find particularly helpful?”	Specialized therapies
Gaps and wishes	“What support would you like?” “What deficits are there in the existing range of treatments?”	Areas with the largest health care gaps

### Data analysis

The systematic analysis of the data was conducted using qualitative content analysis according to Mayring [34], supported by the software program MAXQDA 2022 for analyzing the transcripts.

Categories and coding were based on the topics of the interview guide. The coding was initially carried out through communicative validation with the help of several independent coders at each location. The categories and the respective coding rules were also further developed and refined through a feedback loop. After several exchanges in the study group, categories were adapted and expanded. The classification of symptoms is based on the German S1 guideline long/post-COVID syndrome [32] and the NICE guideline [35]. The classification and analysis of coping strategies is based on the Transactional Stress Model according to Lazarus and Folkman [36] and the Coping Scale COPE according to Carver [37]. The coding was then discussed and compared within and between the locations. Category formation was primarily deductive but inductively supplemented. Demographic characteristics were collected using an additional questionnaire and analyzed using SPSS Version 29.

### Ethics

Before the individual interview started, we obtained consent with a study information letter from all participants. After transcribing the audio files, we anonymized the transcripts and removed all identifying information. Each participant received an identification code with which the source of a quote could be identified. This study received ethical approval from the ethics committee of the Otto-von-Guericke-University Magdeburg (sign 60/22) and the Technical University of Munich (sign 2022−196 S-SR). All interview participants gave both oral and written consent and a physical or electronic signature was obtained. This study was conducted in accordance with the Helsinki Declaration of the World Medical Association [38].

## Results

### Sample characteristics

The majority of interviews (92.5%) were conducted by telephone with people suffering from Long COVID aged 18 and above who did not present with severe mental illness. Forty individual interviews were conducted in total, n = 15 in Magdeburg and n = 25 in Munich. Demographic information of the participants is shown in Table 2. Interview sessions lasted around 37 minutes on average (range 14.5–94 min). Eighty percent of participants are women (n = 32). The average age is 46.9 years (SD = 12.2; range 26–81) and most of the participants (57.5%) have an intermediate level of education according to the CASMIN Educational Classification [39].

**Table 2:** Demographic characteristics of study participants.

Demographic characteristic	Sample Interview study *n = 40*
Gender, n (%)	
Male	8 (20.0)
Female	32 (80.0)
Age, mean (SD) min – max	46.9 (12.2) 26 - 81
Education (CASMIN), n (%)	
high	13 (32.5)
medium	23 (57.5)
low	4 (10.0)
Marital status, n (%)	
Single, no permanent partnership	4 (10.0)
Permanent partnership	9 (22.5)
Married	23 (57.5)
Divorced/ living separately	3 (7.5)
Widowed	1 (2.5)
Employment status, n (%)	*n* = 36
Certified sick	19 (52.8)
Weeks off sick Mean (SD) min – max	*n* = 18 34.8 (26.0) 1 - 82
Medical treatment of COVID-19, n (%)	
None	18 (45.0)
Outpatient	16 (40.0)
Inpatient	1 (2.5)
Inpatient, intensive care unit	5 (12.5)
Call minutes total	1513
Mean (SD)	37.7 (19.2)
min – max	14.5 - 94

*Note:* SD = Standard Deviation. Educational qualifications were classified according to CASMIN Educational Classification [39].

According to self-reports, 30 participants were infected once, while 10 participants reported two previous SARS-CoV-2 infections. Three participants reported their first infection right at the start of the coronavirus pandemic in February, March and April 2020. The majority of initial infections (n = 25) were reported during the second lockdown (October 2020 to April 2021). Reinfections are almost all in the period starting from February until July 2022. Forty-five percent of respondents did not receive any medical treatment for their SARS-CoV-2 infection, forty percent received outpatient treatment and twelve percent were treated in intensive care. Out of 37 cases, 51.4% were vaccinated at the time of infection. Approximately forty-seven percent rate their current state of health as average, none of the respondents as very good, 15% as good and 37.5% as poor to very poor. Almost fifty-three percent of the employed participants were on sick leave at the time of the interview with an average of 34.8 weeks and a maximum of 82 weeks (1.5 years) of sick leave. Nineteen respondents reported a pre-existing physical condition, including tinnitus, migraines, asthma, hypertension, osteoarthritis, Hashimoto’s disease and cancer. Twelve respondents provided information about previous mental health conditions in the interview, five of which explicitly stated conditions including burnout or depression. In the questionnaire, 23 people (57.5%) indicated a current or past mental health treatment.

### Word frequencies

**Fig 1:**
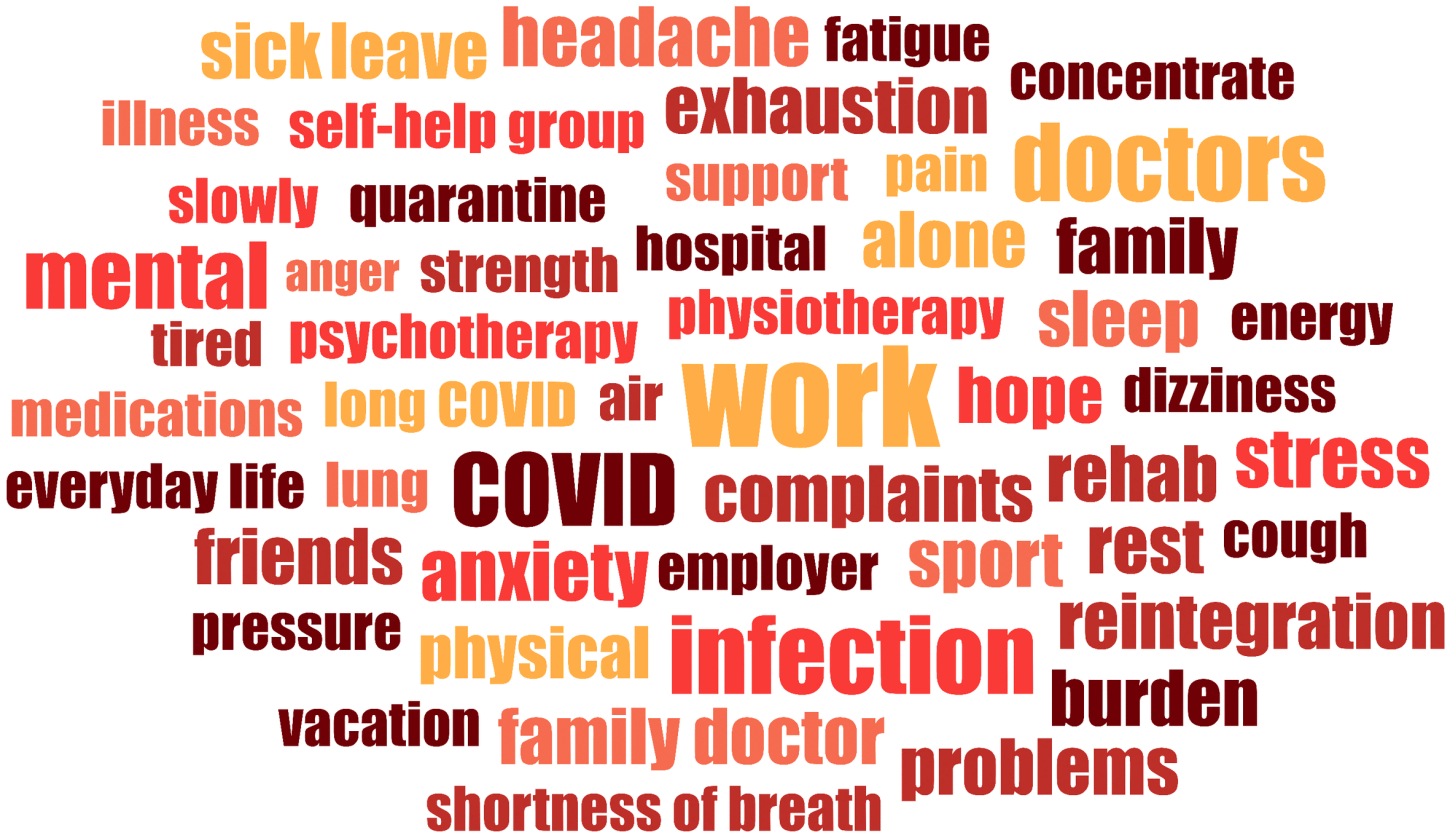
Word frequencies of study participants (ranked 1-50).

At the beginning of the qualitative analysis, we reviewed the most frequently mentioned terms to gain insight into what participants considered most important to share regarding their illness. This allowed special topics to be highlighted or to put more emphasis on them in the further analysis. Statements of the interviewers were removed from all transcripts and only statements of the interviewees were combined. Fig 1 shows the most frequently mentioned terms (ranked 1–50). The minimum frequency of each word was four and related words were combined into one main term. For example, the terms *to work*, *worked*, *job* and *profession* were combined under the term “work”. The terms *to rest*, *resting* and *resting phase* were merged under the term “rest”. Excluded words were pronouns, conjunctions, articles, numbers and words that cannot be interpreted without context. Terms such as *work*, *doctors*, *COVID*, *infection*, *rehab*, *rest*, *family* and *friends* were mentioned most frequently. In terms of complaints *anxiety*, *sleep*, *mental*, *stress*, *exhaustion* and *headache* were often talked about. Work and work-related issues seem to be a relevant topic for those affected, as they were mentioned very frequently. Medical care also seemed to play a major role, as well as individual complaints.

### Symptom overview

**Fig 2:**
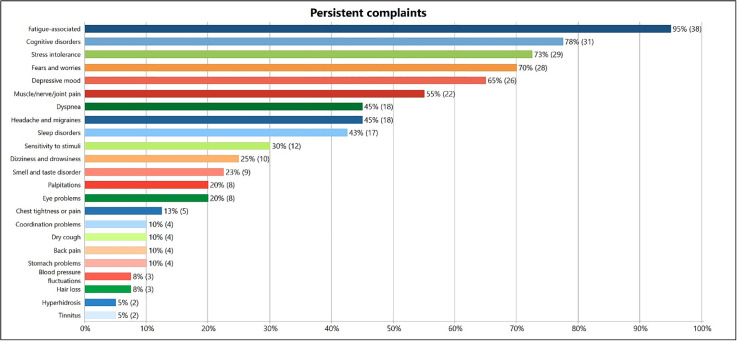
Overview and frequency of reported persistent symptoms.

The following analyses and results only refer to symptoms that were reported as persistent after a SARS-CoV-2 infection. The course of the acute infection was also surveyed. Symptoms experienced during an acute infection, such as fever or sore throat, were not included or evaluated here. We created 23 categories in the section of “persistent complaints and symptoms” – shown in Fig 2 – based on the German S1 guideline long/post-COVID syndrome and the NICE guideline. Thirty-nine out of forty participants had persistent complaints at the time of the interview, only one respondent reported persistent complaints after a SARS-CoV-2 infection, but no ongoing symptoms at the time of the interview. Sixteen respondents commented on the course of their persistent symptoms describing them as undulating or “varying from day to day”.

*Fatigue-associated complaints* as well as *cognitive disorders*, *stress intolerance* and *fears and worries* are reported particularly frequently. To the category of *fatigue-associated complaints,* we added fatigue/tiredness, an increased need for sleep or rest and lack of ability to recover, exhaustion (rapid, severe, extreme, permanent), weakness, lack of drive and lack of energy. *Stress intolerance* could also be included in this category, but it was mentioned so often and since it is one of the more specific symptoms of Long COVID, we counted it as a separate category. *Stress intolerance* includes loss of (physical and/or mental) resilience, reduced or loss of physical performance and activity restrictions. We included cognitive impairments such as word-finding difficulties, forgetfulness, concentration problems/difficulties and brain fog in the category *cognitive disorders*. In the category *fears and worries,* fears of the future and uncertainty, professional worries and insecurities, financial worries and the fear of re-infection were mentioned above all.

Other common complaints are *depressive mood*, like sadness, lack of joy, loss of interest, mood swings, mind wandering, helplessness or feelings of worthlessness; *muscle, nerve and joint pain*; *headaches and migraines*; *dyspnea* (shortness of breath or breathing difficulties); *sleep disorders* (insomnia, difficulty falling asleep or staying asleep); *sensitivity to stimuli* (noise or light); *dizziness and drowsiness*; *smell or taste disorder*; *palpitations*; *eye problems* (eye pressure, watery eyes, vision deteriorated); *chest tightness or pain*. Less frequently mentioned complaints (<5) are *coordination problems*, *dry cough*, *back pain*, *stomach problems*, *blood pressure fluctuations*, *hair loss*, *hyperhidrosis* and *tinnitus*.

When asked which complaint causes the most distress, twenty-two participants named tiredness or exhaustion, six named *stress intolerance*, four named *muscle and joint pain*, and also four named *fears and worries*. These results are also consistent with the most common symptoms from the German S1 guideline long/post-COVID syndrome and the NICE guideline and confirm the high incidence of *fatigue-associated complaints* and *cognitive disorders.* Mental complaints such as *fears and worries* and *depressive moods* are also very important to focus on, as these can have a major impact on the quality of life.

### Additional individual stressors

In addition to the physical and mental complaints, further stressors and burdens were identified. Frequently mentioned stressors were major symptom-related and professional insecurities, financial worries, uncertainty and worries about the future.

“*Long COVID - will it stay like this for the rest of my life? Will it stay like this for another year, two years? No one can tell me. And that’s what actually bothers me the most, the fact that I don’t know when it will go away, whether it will go away at all.”* (ID105)

In addition, withdrawal from social life, lack of acceptance and lack of understanding are reported. Respondents feel that they are not taken seriously and feel left alone.

“*My acquaintances and friends all say:* ‘*Long COVID doesn’t really exist and you’re not really sick. And if you just lie around, you won’t get better. You have to do more than that*’*. Besides, I also had a general practitioner who was of the same opinion.”* (ID124)

This includes the issue of stigmatization, which is often reported on. Reproaches from others make those affected feel labeled or put down as malingerers. They feel discriminated against and excluded.

“*Because you’re often portrayed as a malingerer or:* ‘*You’re just telling yourself that or it’s not that bad and you’ll be fine, you just have to... you just have to pull yourself together*’.” (ID01)

A great stressor is the loss of the “old life” and the loss of identity and independence. Those affected feel “torn out of life”. They see themselves as a burden. Problems within the healthcare system, such as long waiting times for specialists, the lack of suitable contact persons and the lack of therapy or treatment in general are also reported as stressors. Participants would like to see more education about Long COVID in society and specifically among physicians.

“*Because they say:* ‘*I just can’t imagine what you actually have*’*. You can’t see it. You can see a broken leg. You can’t see COVID and you certainly can’t see Long COVID.”* (ID16)

All of the stressors mentioned have an impact on the everyday, social and professional life and are reflected in a lack or loss of quality of life for those affected.

“*And the doctors you go to always tell you not to be like that, that they can’t find a cause and that it’s just your imagination. That I simply don’t want to go to work. But yes, I’d actually be happy if I could go to work. Well, I do go to work, but that also means that by going to work, I no longer have a life, not at all.”* (ID11)

The named needs and requirements of the participants are understanding, recognition and support, more visibility and education about Long COVID, participation in social life or return to “normal life” and a central point of contact. Those affected have to cope not only with persistent physical and mental symptoms, but also with the additional burdens that this disease entails.

### Coping strategies

Participants were asked how they usually deal with their complaints and what has worsened or improved symptoms so far. A worsening of the symptoms was reported due to (over)exertion and (over)strain, stress, sporting activity, excitement and conflict, heat, noise and alcohol consumption. Improvements were achieved through rest, relaxation and sleep, retreat, planning activities, taking breaks, minimal exercise, yoga and meditation, physiotherapy and exchange with others.

“*I talk a lot about this kind of thing with my husband or with my sister or friends [...]. The communication itself actually helps me quite a lot.”* (ID16)

When asked what helped most in dealing with the long-term effects, the most common answers were rest, acceptance, family support, self-help, taking care of oneself and talking about it.

“*I think what would help the most, but what I can’t quite do yet, would certainly be acceptance. Accepting the illness. Accepting that it is an illness and that you don’t know how long it will last, whether it will last and how it will develop. And just […] accept it as it is.”* (ID117)

The interviews were analyzed based on the Transactional Stress Model according to Lazarus and Folkman and the Coping Scale COPE according to Carver. According to the Transactional Stress Model [36], there is a stimulus or stressor at the beginning. Here it is the long-term consequences of the SARS-CoV-2 infection (number and severity of symptoms and other burdens). This is followed by the primary evaluation or interpretation of the stressor through the perception filter. If this evaluation leads to “dangerous”, involving challenges, threats or losses, as is the case for most Long COVID sufferers, a secondary evaluation will follow, analyzing the available resources. According to the Transactional Stress Model, a lack of resources leads to stress (e.g., the absence of cure for Long COVID, a lack of therapy or restrictions due to complaints). Stress management or coping can then be problem-oriented with behavioral strategies (this involves changing the situation itself through action-oriented, active coping, whereby the stressor is addressed directly) or emotion-oriented with cognitive strategies (this involves changing the relation to the situation through cognitive coping, whereby the stressor itself cannot be changed). This can be followed by a reassessment with adaptation and learning. The Brief COPE [37] is the most frequently used coping scale [40]. We combined the Transactional Stress Model with the terms of the COPE scale and categorized them according to adaptive (beneficial) and maladaptive (harmful) strategies, because Carver himself did not divide his scale into emotion-oriented and problem-oriented coping. As we were able to find further coping strategies or attempts in the interviews, we used terms from other questionnaires on coping, for example the *Essen Coping Questionnaire* [41] or *The Bernese Coping Modes* [42] and also added own terms. Our results can be found in Table 3.

**Table 3:** Results of coping strategies mentioned by the study participants (n = 40).

Coping strategies			
Emotion-oriented		Problem-oriented	
Adaptive	Maladaptive	Adaptive	Maladaptive
Emotional support (35) Self-care (16) Emotional distraction (10) Positive thinking (9) Acceptance (9) Hope (7) Fighting spirit/persevere (7) Express/allow emotions (5) Relativization (1)	Repression/avoidance of emotions (11) Denial/lack of acceptance (3) Self-blame/ self-depreciation (3)	Planning/Pacing (22) Physical activity/ movement (18) Self-help (14) Instrumental/social support (12) Action-oriented distraction (3)	Withdrawal/distance (14) Use of medication (7) Avoidance (7)

*Note:* The number of participants for whom the respective strategy was coded is shown in brackets.

Almost all respondents (n = 35 out of 40) mentioned *emotional support* as a coping strategy. We included everything that can have a positive effect on the mental state in this category. For example, family support, talking to others (including self-help groups), attention/understanding and encouragement from friends/family/professionals or pets, relaxation techniques and meditation. On the problem-oriented side *planning/pacing* was mentioned most frequently as a strategy (n = 22). This includes daily or activity planning, taking breaks and adhering to boundaries.

Other frequently mentioned emotion-oriented adaptive strategies are *self-care* (think about yourself, take good care of yourself, look after yourself and your body), *emotional distraction* (distract yourself mentally, thinking about other things), *positive thinking*, *acceptance*, *hope*, *fighting spirit/persevere* and *acting out/allow emotions*. We found *repression and avoidance of emotions* as emotion-oriented and maladaptive strategy in eleven respondents.

*Physical activity/movement*, *self-help* (e.g., searching for information, trying out alternatives, taking the initiative and seeking help*)* and *instrumental/social support* (practical help, for example in the household or with childcare) are other adaptive problem-oriented strategies mentioned. Maladaptive strategies here are (social) *withdrawal/distancing*, *use of medication* (especially pain medication with a risk of dependency in the long term, independently taken or taken without physician’s advice) and *avoidance* (of social contacts and events).

There is an interplay between emotion-oriented and problem-oriented as well as adaptive and maladaptive coping among our participants. If respondents do not cope in a problem-oriented way, at least one emotion-oriented strategy was mentioned.

We have also calculated some correlations. Almost ninety-four percent of women cope with *emotional support*, but also 62.5% of men. Fifty-nine percent of women (compared to 37.5% of men), 69% with a high level of education and 25% with a low level of education cited *pacing* as a strategy. More women than men (25% to 12%), 38% with a high level and nobody with low level of education stated *positive thinking*. Twenty-eight percent of women, but no men, mentioned *acceptance*. Twenty-one percent of women, but no men, show *fighting spirit*. People who were treated as inpatients did not mention *positive thinking* or *acceptance*. Both women (34%) and men (37%) and people with a very poor assessment of their health status were more likely to withdraw. Twenty-one percent of women and no men stated that they take pain medication (*p* >.05 in each case).

## Discussion

The data obtained from the interview process showed that long-term physical complaints were the focus of respondents’ concerns. When asked about previous mental illnesses and complaints after COVID-19, some respondents answered cautiously or initially did not mention any complaints. This could be due to earlier stigmatization and partial psychologization of the disease and could have led to an underestimation of previous mental illnesses. However, later during the interview the topic of fears and worries or other mental impairments did come up. We coded *fears and worries* for 70% of respondents and *depressive mood* for 65%. In addition to the high physical stress, this also shows a considerable mental stress of the interviewees. The frequently reported individual stressors also highlight the importance of mental stress caused by Long COVID or suggest a high level of mental stress and places the focus on the interplay between body, mind and social components. Therefore, clinicians should consider the elevated risk of developing depression, anxiety disorders or Somatic Symptom Disorder (SSD) in patients with Long COVID. Especially SSD could be a less stigmatizing diagnosis for these patients since the psychological burden through somatic symptoms is addressed in this diagnosis. According to a recent study, SSD could play a crucial role in psychological burden in patients with persistent symptoms after COVID-19 [43]. This indicates that, in addition to biological targets, treatment options on a psychological and social level are also required [13,15,44].

Another frequently mentioned topic of concern was work and working life. Important issues were ability to work, reintegration and financial concerns. Interviewees often wanted to be able to return to work. Once they were able to return to work, their energy was only sufficient for their job duties. According to the German Social Code, a person can be considered “long-term sick” if they are ill for longer than six weeks, as the normal continued payment of wages by the employer ends and sickness benefit is paid for a maximum of 78 weeks sick. The mean number of weeks on sick leave among our respondents was 34.8 weeks. Fifteen of the employed participants (83%) were on sick leave for more than 6 weeks. One respondent had already been on sick leave for over 78 weeks. This shows the economic significance, that Long COVID can become a chronic illness and is a burden not only to those affected, but also a major challenge for the economic system. Other studies have shown the significant financial burden with associated health anxiety and considerable healthcare costs of Long COVID [10]. Financial resources were identified as one of the biggest challenge in dealing with Long COVID [12,25].

The results indicate that *emotional support* was most frequently used as an adaptive emotion-oriented coping strategy. This supports the results of previous studies, in which coping strategies regarding Long COVID and the pandemic mainly focused on emotional strategies and social support [19,20,28,29]. Emotion-oriented coping, such as efforts to reduce or manage emotional distress, is primarily used for chronic conditions and uncontrollable stressors where “nothing can be done” or the situation itself cannot be changed [37]. This also applies to Long COVID – there is no cure, the causes are not yet fully understood, and treatment options are limited. Emotion-oriented coping should therefore be strengthened or combined with problem-oriented strategies at the action level, such as pacing. Pacing could also be a promising strategy, as it is currently considered the most effective strategy for dealing with stress-induced symptom worsening (post-exertional malaise) [45]. Our data shows that only 25% of participants with a low level of education named pacing as a strategy. A more comprehensive clarification about Long COVID and psychoeducation, as well as providing information to increase awareness of Long COVID should therefore be encouraged here. Emotional support, especially from family and friends, conversations and exchanges with others who have experienced similar issues, understanding and compassion seem to play an important role in coping with the illness. Emotional support is, therefore, a fundamental resource if other emotional resources such as hobbies, sports or social contacts are lost (during lockdown) or can no longer be used to the same extent (due to physical impairments). The gain or loss of resources can considerable influence the way in which people cope with stress. This is why it is important to make further resources visible and encourage those affected to use them.

Looking ahead, these coping strategies outlined here may be helpful not only for people affected by Long COVID, but also for those affected by other post-viral phenomena or those affected by conditions with persistent symptoms. Adaptive coping is aimed at resilience and sustainable coping with stress. In cases of illnesses with a physical cause, mental support should also be a focus. In addition, the stigma surrounding “invisible diseases” should be reduced. Even if the burdens are not immediately visible from the outside, it does not mean that they are not real or that they should be played down. The perceived stigmatization of their “persistent” symptoms by the participants was a significant barrier to adopt the coping strategy of social support [25]. Understanding and being taken seriously is an important emotional support for those affected. Further long-term research on symptoms, stressors and coping with them is needed to consolidate the importance of emotion-oriented strategies and to ensure that those affected also report an improvement in symptoms after a longer period of time.

## Limitations

The potential for regional or contextual differences is one limitation of the study. Some cities and regions are rather underserved due to the lack of Long COVID outpatient clinics, whereas in others care is better organized. Another limitation is the respondents’ self-reporting of SARS-CoV-2 infections and testing. Since we did not see nor examine the respondents in person as part of the interview study and did not request any evidence, we can only refer to self-reported infections here.

When categorizing the symptoms, it was not always possible to make a clear distinction between the complaints. For example, *fatigue-associated complaints* and *depressive symptoms* overlap to some extent and *stress intolerance* could also be counted to *fatigue-associated complaints*.

The used coping scales were not exhaustive. Carver’s scale, therefore, had to be adapted conceptually in some cases, e.g., Carver’s “substance use” did not appear in our interviews, but “use of medication” without a named dependency did. Several coping strategies from Carver did not appear explicitly in our interviews, e.g., “humor” or “religious coping”. Other scales contained many terms, but no division into emotion-oriented and problem-oriented strategies. Lazarus and Folkman, themselves, also did not use any direct terms for coping strategies. In later publications, terms such as *distance*, *social support* or *planned problem solving* were added. It should also be mentioned that some participants initially rated *use of medication* and *withdrawal* as a positive (adaptive) strategy to avoid worsening of symptoms, but later reflected that they feared side effects with long-term use of medication and that social withdrawal is not conducive to their social life in the long term. The distinction between some coping strategies is also not entirely clear-cut. For example, *self-help* or *physical activity* also have a positive mental effect. However, we were primarily concerned with active coping, that something is actively done or undertaken. In the case of emotion-oriented coping, it was primarily about the cognitive added value. In some examples, *distraction* was described in terms of cognition and action, hence the assignment to both strategies. *Positive thinking* and *hope* have been categorized as separate strategies, as both were described differently.

## Conclusion

The prevalence of SARS-CoV-2 infections has decreased and the pandemic is considered to be officially over. However, the long-term consequences have not yet been overcome and will continue to affect and burden many of people affected by long-term consequences of COVID-19. Those affected report many different complaints and stressors on a physical, mental and social level. The complaints and stressors, thus, have a major impact on their quality of life. Dealing with the disease remains difficult due to insufficient research into the causes and lack of adequate therapy. A psychosomatic-psychotherapeutic intervention was developed within the *PsyLoCo* study, among others based on this qualitative interview study, to provide support for psychosocial needs of people affected by Long COVID. The most salient stressors mentioned were the persistent symptoms and other individual burdens associated with the illness. The most frequently mentioned persistent symptoms were fatigue-associated complaints, cognitive disorders, stress intolerance, fears and worries. Individual stressors included work-related issues, such as job insecurity and financial worries, withdrawal from social life, lack of acceptance and lack of understanding, stigmatization, loss of the old life, loss of identity and independence. Participants wished for more opportunities to deal with their illness, more acceptance and recognition and better support, especially when returning to social and working life. In our sample, these burdens were most coped by emotional support, pacing, physical activity, self-care, self-help and withdrawal. It is emphasized that emotion-oriented coping should be strengthened in the case of long-term illnesses and that emotional support is a fundamental resource for coping with an illness. This can be useful for other post-viral diseases. The results obtained could facilitate a more comprehensive understanding of the individual coping patterns employed by individuals when dealing with post-viral phenomena in cases of COVD-19. The primary focus should be on providing support to those affected, with the aim of reducing the burden of the disease, enhancing their quality of life and facilitating their reintegration into social life.

## Abbreviations

NICE: National Institute for Health and Care Excellence
SSD: Somatic Symptom Disorder
SARS-CoV-2: Severe Acute Respiratory Syndrome Coronavirus type 2
WHO: World Health Organization.
